# Surface Stabilization Affects Toxicity of Silver Nanoparticles in Human Peripheral Blood Mononuclear Cells

**DOI:** 10.3390/nano10071390

**Published:** 2020-07-17

**Authors:** Barbara Vuković, Marija Milić, Blaženka Dobrošević, Mirta Milić, Krunoslav Ilić, Ivan Pavičić, Vatroslav Šerić, Ivana Vinković Vrček

**Affiliations:** 1 Department of Clinical Laboratory Diagnostics, University Hospital Osijek, Josipa Huttlera 4, 31000 Osijek, Croatia; barbara.simonovic@gmail.com (B.V.); marija.milicgall@gmail.com (M.M.); dobrosevic.blazenka@kbo.hr (B.D.); seric.vatroslav@kbo.hr (V.Š.); 2 Faculty of Medicine, Josip Juraj Strossmayer University of Osijek, Josipa Huttlera 4, 31000 Osijek, Croatia; 3 Institute for Medical Research and Occupational Health, Ksaverska cesta 2, 10000 Zagreb, Croatia; mmilic@imi.hr (M.M.); kilic@imi.hr (K.I.); ipavicic@imi.hr (I.P.)

**Keywords:** nanosafety, surface coating, cellular uptake, apoptosis, oxidative stress, genotoxicity

## Abstract

Silver nanoparticles (AgNPs) are one of the most investigated metal-based nanomaterials. Their biocidal activity boosted their application in both diagnostic and therapeutic medical systems. It is therefore crucial to provide sound evidences for human-related safety of AgNPs. This study aimed to enhance scientific knowledge with regard to biomedical safety of AgNPs by investigating how their different surface properties affect human immune system. Methods: preparation, characterization and stability evaluation was performed for four differently coated AgNPs encompassing neutral, positive and negative agents used for their surface stabilization. Safety aspects were evaluated by testing interaction of AgNPs with fresh human peripheral blood mononuclear cells (hPBMC) by means of particle cellular uptake and their ability to trigger cell death, apoptosis and DNA damages through induction of oxidative stress and damages of mitochondrial membrane. Results: all tested AgNPs altered morphology of freshly isolated hPBMC inducing apoptosis and cell death in a dose- and time-dependent manner. Highest toxicity was observed for positively-charged and protein-coated AgNPs. Cellular uptake of AgNPs was also dose-dependently increased and highest for positively charged AgNPs. Intracellularly, AgNPs induced production of reactive oxygen species (ROS) and damaged mitochondrial membrane. Depending on the dose, all AgNPs exhibited genotoxic potential. Conclusions: this study provides systematic and comprehensive data showing how differently functionalized AgNPs may affect the human immune system. Presented results are a valuable scientific contribution to safety assessment of nanosilver-based blood-contacting medical products.

## 1. Introduction

In today’s nano-world, two ‘ologies’ are constantly opposed: nanotechnology and nanotoxicology—one is trying to convince public about the usefulness of nanomaterials (NMs), while the other is trying to warn about potential nano-hazard on human health. The European nanoproduct inventory estimated that the highest amount of all nano-enabled products contains silver and half of these silver-containing nanoproducts are claimed to exhibit biocidal characteristics [1]. One of the most prominent and promising field for the application of silver nanoparticles (AgNPs) is the healthcare sector affected by hospital acquired infections [2]. Indeed, AgNP are already used in many different medical devices, such as dental implants and prostheses, catheters, endotracheal tubes, wound dressing materials, etc. [3]. Lately, AgNPs proved to have other interesting characteristics for nanobiomedicine applicability, like nanovectors for vaccines, drug-delivery systems and diagnostic platforms [4,5]. The COVID-19 pandemic crisis highlighted another important area where AgNPs biocidal properties could be deployed to develop new tools and treatments for safeguarding global health against ever-mutating deadly viruses. The ability of antiviral activity of AgNPs was demonstrated on viruses such as HIV or rhinovirus [6], hepatitis B [7], respiratory syncytial virus [8], or herpes simplex virus type 1 [9]. However, it is well known that biomedical application of any metal-based nanosystems is a “double-edged sword“. In the case of AgNPs, strong oxidative activity and release of very toxic Ag ions may lead to oxidative stress induction in human cells and tissues through production of reactive oxygen species (ROS) and depletion of mitochondrial membrane potential (Δψm), representing initial proapoptotic signal [10,11]. Consequences of such oxidative stress may be numerous such as cytotoxicity, programmed cell death, genotoxicity, activation or supression of immune system [12,13,14]. It has been evidenced that AgNPs toxicity effects depend on their physico-chemical properties, such as size, surface charge, type of surface stabilization, solubility [8,9,10,11,12]. For successful development of efficient and safe AgNPs-enabled medical devices and biocidal products, it is of paramount significance to understand how different physico-chemical properties of AgNPs govern their interaction with the immune system. Circulating human peripheral blood mononuclear cells (hPBMC) are of particular interest as main representatives of human immune system and the most suitable cell population for immunocompatibility assays. According to the ISO 10993-4 guidelines, all medical devices that may come into contact with circulating blood has to fulfill biocompatibility requirements evaluated by hematologic, coagulation and immunologic tests [13,14,15]. Despite increasing number of silver nanoproducts in the healthcare sector, science-based data on complex interactions of AgNPs with the immune system are still lacking or being poorly understood. Literature search in the ISI Web of Science (WoS) database performed in January 2020 for the terms “nano*” AND “silver*” resulted in total number of 95,259 publications, while the terms “nano*” AND “silver*” AND “immun*” OR “lymph*” OR “monocyt*” gave only 2987 publications. From these publication pool, only small number of studies reported comprehensive evaluation of interaction between AgNPs and hPBMC. Main outcomes of these studies are presented in Table S1 of Supporting Materials.

This study aimed to evaluate comprehensively how different physico-chemical properties of AgNPs govern their uptake, cytotoxicity and genotoxicity effects in hPBMCs. Four different types of AgNPs were used considering the impact of surface stabilization and surface charge on a dose- and time-response of hPBMC. The AgNPs were designed to be of 10 nm size and characterized by neutral, positive and negative surface charge. For this purpose, neutral polymer poly(vinylpyrrolidone) (PVP), positively charged ε-poly-L-lysine (PLL) and negatively charged bis(2-ethylhexyl) sulfosuccinate sodium (AOT) were used for AgNPs stabilization. In addition, AgNPs stabilized with bovine serum albumin (BSA) were included to evaluate the impact of protein corona formed on the AgNPs surface. Coating agents (PLL, PVP and AOT) and ionic Ag (in the form of AgNO_3_) were also included in the study to test the effect of coating agents themselves and the effect of potentially released Ag ions, respectively. Interaction of AgNPs with hPMBC was investigated by means of AgNPs cellular uptake and their ability to trigger cell death, apoptosis and DNA damages through induction of oxidative stress and damages of mitochondrial membrane. Whole study was performed on freshly prepared hPBMCs to prove its suitability for hemocompatibility testing of nanomaterials. 

Design of this systematic and comprehensive study allowed confirmation of hypothesis that safety of AgNPs significantly depends on their surface properties. Obtained results represent valuable contribution to the current state of knowledge regarding the effects of biocidal nanomaterials on the immune system and promote implementation of the safe-by-design concept in research and development of nano-enabled medical devices as early as possible [16].

## 2. Materials and Methods 

### 2.1. Preparation of hPBMC

The whole study protocol was approved by the ethical committee of University Hospital Osijek and Faculty of Medicine, University J.J. Strossmayer of Osijek. Whole blood was collected in vacutainers with EDTA-anticoagulant from six healthy volunteers that were non-smoker, non-pregnant, free of medications for the last 10 days and characterized by the absence of leukocytosis or leukopenia in blood and with normal percentage of lymphocytes and monocytes in differential blood count. Fresh whole blood samples were immediately used for isolation of hPBMCs that were isolated using Lymphoprep (Axis-Shield, Oslo, Norway). Briefly, whole blood samples were first diluted by addition of an equal volume of 0.9% NaCl and transferred in centrifuge tubes filled with Lymphoprep solution. After centrifugation at 800× *g* for 20 min at room temperature in a swing-out rotor without break, the hPBMCs formed a distinct band in the middle of the sample. Cells were harvested with Pasteur pipette, diluted with 0.9% NaCl and pelleted by centrifugation at 250× *g* for 10 min. After discarding the supernatant, cells were dispersed in PBS. Both the whole blood and isolated cells were counted on an automated hematology analyzer (Sysmex XN 2000, Sysmex, Kobe, Japan). A number of isolated hPBMCs was adjusted to the 10^6^ cells/mL with PBS. In this media (PBS + hPBMC), total protein content was quantified by Micro Total Protein Kit (Merck, Darmstadt, Germany). 

### 2.2. Synthesis and Characterization of AgNPs

The syntheses of AgNPs with different surface coatings were conducted by reducing silver nitrate with sodium borohydride in the presence of AOT, PVP and PLL as coating agents as previously described [17]. The formation of nano-sized silver particles was verified by the presence of a surface plasmon resonance (SPR) peak measured using an UV-Vis spectrophotometer (CARY 300, Varian Inc., Australia). Careful characterization of each AgNP type was conducted in ultrapure water at the concentration of 10 mg Ag/L. The size and charge of AgNPs were measured at 25 °C by dynamic (DLS) and electrophoretic light scattering (ELS), respectively, at 173° using Zetasizer Nano ZS (Malvern, UK) equipped with a green laser (532 nm). The DLS and ELS data were processed by the Zetasizer software 6.32 (Malvern Instruments, Worcestershire, UK). Particles were visualized using a transmission electron microscope (TEM, Zeiss 902A, Jena, Germany). Released free Ag ions fraction in each AgNPs was determined by ultrafiltration across a membrane of 3 kDa Amicon-4 Ultra centrifugal filter units (Merck Millipore, Darmstadt, Germany). Details on synthesis and characterization are given in the Supplementary Information file.

Dissolution and agglomeration behavior of AgNPs were evaluated in the following media: UPW, phosphate buffer saline pH = 7.4 (PBS) and PBS containing hPBMC. In each media, AgNPs were incubated for 1 h at a concentration of 10 mg Ag/L and *d*_H_, ζ potential and released Ag^+^ were measured as described above. In the case of PBS containing hPBMC, DLS and ELS experiments did not provide reliable data as the presence of cells increased significantly light scattering and shielded signals from AgNPs. In this case, dispersion of AgNPs were inspected visually, while protein corona formation was confirmed following protocol as described elsewhere [18].

### 2.3. Treatment of hPBMC with AgNPs

For the treatment of hPBMC, stock solutions of AgNPs, coating agents and AgNO_3_ were prepared in ultrapure water. The hPBMC were treated with AOT-, PVP- and PLL-AgNPs at concentrations of 1, 5, 10 and 25 mg Ag/L, while treatment with BSA-AgNPs was in the range between 0.2 and 10 mg Ag/L due to their higher toxicity. After incubation with AgNPs for 1 or 3 h at 37 °C in the dark, cells were washed two times with PBS to remove the AgNPs, centrifuged at 250× *g* for 5 min and prepared for further analysis by flow cytometry or confocal and light microscopy. In addition to AgNPs, effects of ionic Ag (in the concentration range of 0.01–1 mg Ag/L) and surface coating agents were also studied. Selected concentration range for ionic Ag was based on the data obtained by dissolution experiments of AgNPs that showed Ag^+^ release from the AgNPs surface below 1.0%. Applied concentrations of PVP, PLL and AOT (0.3%, 0.00005% and 0.01 mM, respectively) corresponded to the levels of coating agent present in the respective AgNPs dispersion at the highest concentration applied to the hPBMC (25 mg Ag/L). Effect of BSA was not evaluated because it was reported that BSA-based NPs are safe and hemocompatible [19]. All treatments were performed for isolated hPBMC of each volunteer.

### 2.4. Flow Cytometry Experiments

Evaluation of cell death, apoptosis induction, oxidative stress response and particle uptake in treated hPBMC was performed by flow cytometry using FACS Calibur FCM (BD Bioscience, San Jose, CA, USA). Configuration of this flow cytometer consists of one laser (argon, ex. 488 nm) and three PMT detectors of fluorescence with band-pass (BP) and long-pass filters (FL1 530/30 BP, FL2 585/42 BP and FL3 650 LP). The forward scatter (FSC) signal was collected by the FSC diode. It is the light scattered at narrow angle and represents the size of the cell. The side scatter (SSC) signal was collected by the 90° collection lens and represents cellular inner complexity. Optimization of instrument settings including voltages, amperes and compensation of detectors was performed with CaliBRITE beads (BD Bioscience, San Jose, CA, USA) and adjusted to the isolated hPBMCs. Acquisition and analysis of collected data were performed in Cellquest software (BD Bioscience, San Jose, CA, USA). Acquisition counter was set on 10,000 events for all experiments to eliminate debris contribution (residual neutrophils and erythrocytes). FACSCalibur is an analogue system in which optical signals are converted to electronic signals and then to digital values; predetermined instrument settings cannot be changed later during the software data analysis. For that reason, results were normalized. Otherwise, results from different experiments could not be compared.

#### 2.4.1. Evaluation of Cell Death and AgNPs Uptake

Analysis of AgNPs uptake, cell death and apoptosis induction were performed simultaneously in the same experiment evaluating the geometric means of forward scattered (FSC), side scattered (SSC) light and fluorescence signals (FL). The FSC determines cell size whereas SSC depends on the density of the cell. During data analysis, different gating strategies were applied for the uptake and evaluation of live, dead and apoptotic cells. The gating strategy was performed on FSC (linear)/SSC (log) dot-plot (see Figure S1 in the Supporting Materials). Live cells with or without AgNPs uptake retain the same size, while dying cells decrease in size. Cytotoxic effects of AgNPs were evaluated on all acquired hPBMCs, while cell debris was excluded by setting the gate also on dead hPBMCs, which were characterized by decreased FSC and elevated SSC. Uptake of AgNPs was evaluated only on living cells by setting the gate on hPBMCs with the same FSC as in control sample (untreated hPBMCs), but with elevated SSC (live hPBMCs with AgNPs uptake).

Analysis of apoptosis induction by AgNPs in hPBMC was performed using FITC Annexin V Apoptosis Detection Kit I (BD Bioscience Pharmingen, San Diego, CA, USA). This kit combines FITC Annexin V and propidium iodide (PI) staining allowing detection of viable intact cells (Annexin V^−^ and PI^−^), dead cells (Annexin V^−^ and PI^+^), early apoptotic cells with intact membranes (Annexin V^+^ and PI^−^), and sum of late apoptotic, necroptotic and secondary necrotic cells (Annexin V^+^ and PI^+^). While cells stained only by Annexin V or PI are those that are early apoptotic or dead, respectively, double positive staining detects not only cells in late apoptotis, but also cells then undergo necroptosis/programmed necrosis [20].

Treated hPBMCs were resuspended in binding buffer (Hepes/NaOH/NaCl/CaCl_2_). Volume of 5 µL AnnV and PI were added to a 100 µL of hPBMCs (1 × 10^6^ cells/mL) and cells were incubated for 15 min at room temperature in the dark. After incubation, cells were ready for flow cytometry acquisition and analysis. Negative control consisted of unstained, untreated hPBMCs (unstained control), while positive controls were fluorescence-minus-one (FMO) and whole-stained controls. Two different FMO positive controls were used; one was stained only with AnnV and the other only with PI. Whole-stained control was stained with both fluorescent probes. Cells used for positive control were hPBMCs left over night on 37 °C in the dark and acquisition was performed the day after the experiment. Acquisition of cells in negative control was performed together with acquisition of treated cells. Negative, unstained control was used to evaluate a percentage of living cells in the sample prior to the treatment. FMO controls were used to set a proper quadrant markers to separate living cells, early apoptotic cells, late apoptotic (secondary necrotic) cells and dead cells (see Figure S2 in the Supporting Materials). Such strategy was needed as Annexin V and PI double-stained cells form heterogeneous population without clear separation between dead and late apoptotic cells due to a production of apoptotic bodies. Fluorescence signal from Annexin V was detected on FL1 detector and fluorescence signal from PI was detected on FL2 detector (but it also breaches through the FL3 detector) (Figure S2 in the Supporting Materials). 

The geometric means of scattered (SSC) light and fluorescence signals (FL1 and FL2) were obtained for data analysis of uptake and apoptosis induction, respectively. The uptake efficiency of AgNPs was determined by measuring the increase of the SSC, as its intensity is proportional to the intracellular density and granularity of the cells [21]. For the apoptosis induction, results were normalized by mean fluorescence intensity (MFI) that were obtained by dividing geometric mean of the fluorescence intensity of the treated sample by the value of untreated (negative control) sample [21,22]. Measurements were repeated three times for each experiment. All results are presented as the mean of six experiments including standard deviation (SD) represented as error bars.

#### 2.4.2. Evaluation of Oxidative Stress Response

Oxidative stress response to AgNPs treatment was evaluated on the same isolated hPBMCs, but in different experiments using three different fluorescent dyes. The 2’,7’-dichlorofluorescein diacetate (DCFH-DA, Sigma Aldrich, Darmstadt, Germany) was used to detect level of peroxy radicals, while the dihydroethidium (DHE, Sigma Aldrich, Darmstadt, Germany) enabled determination of intracellular superoxide radical (O_2_^−^) production. The 3’,3’-dihexyloxacarbocyanine iodide (DiOC_6_, Sigma Aldrich, Darmstadt, Germany) was used to measure depletion of mitochondrial membrane potential (Δψ_m_) as a consequence of the loss of mitochondrial membrane integrity [10,11].

Stock solutions of DCFH-DA, DHE and DiOC_6_ were used at concentrations of 200 µM, 1 mM and 10 µM, respectively, and diluted with UPW to concentrations of 10 µM, 50 µM and 0.1 µM in working dye solutions, respectively. After adding suspension of treated cells in each working dye solutions, final concentration of DCFH-DA, DHE and DiOC_6_ were 5, 25 and 0.05 µM, respectively. Cells were incubated at 37 °C in dark with DiOC_6_ for 15 min, or with DCFHE and DHE for 30 min. Afterwards, cells were washed with 2 mL of PBS and centrifuged on 300× *g* for 5 min. Supernatant was discarded and 200 µL of PBS was added for FCM acquisition and analysis.

Three different controls were used for these experiments: unstained negative (untreated cells), stained negative (untreated stained cells) and stained positive control (treated with different AgNPs at concentration of 1 mg Ag/L). For positive control, hPBMCs were incubated with 1 µM H_2_O_2_ for 30 min and washed with PBS. After that, washed hPBMCs were stained with DCFH-DA or DHE. Only negative controls were used for DiOC_6_ (unstained and stained).

Fluorescence signals from DCFH-DA (Figure S3 in the Supporting Materials) and DiOC_6_ (Figure S4 in the Supporting Materials) were detected on FL1 detector, while fluorescence signal from DHE was detected on FL2 detector (Figure S5 in the Supporting Materials). Results were normalized by MFI as described above. Measurements were repeated 3 times for each experiment. All results are presented as the mean of 6 experiments including standard deviation (SD) represented as error bars.

### 2.5. Visualization of Treated hPBMC

Immediately after exposure of freshly isolated hPBMC to different AgNPs, cells were centrifuged by Cytospin at 30× *g* for 10 min using cytology slides. Thereafter, slides were fixed with formaldehyde (4% (*v*/*v*) in PBS, Merck, Darmstadt, Germany) for 10 min at −20 °C. Cells were then stained May-Grünwald solution (Kemika, Zagreb, Croatia) for 5 min, after which they were placed in sterile PBS for 2 min. Slides were subsequently submerged in Giemsa solution (Kemika, Zagreb, Croatia), diluted 1:20 in MilliQ H_2_O, for 20 min. Cells were then visualized by light microscopy to evaluate morphological changes.

Cellular uptake of AgNPs was inspected by confocal laser scanning microscope (CLSM) using reflection contrast mode [23]. Cells were seeded on cover slips placed in 12-well plates (5 × 10^3^ cells/well) and allowed to attach for 24 h. The cell culture medium was then removed and cells were exposed to AgNPs at concentration of 1 mg Ag/L. After incubation for 3 h, cells were thoroughly washed 2 times with PBS and treated with ice-cold methanol (−20 °C) for 3 min. Cells were then treated with 0.1% Triton X-100 solution in PBS for membrane denaturation and again washed with sterile PBS. Fluorescein Isothiocyanate-Labeled Phalloidin (Sigma Aldrich, Darmstadt, Germany) staining was performed by adding 1 µg/mL of antibody solution in sterile PBS, followed by 20 min incubation. Excess dye was washed with PBS. Next, Hoechst dye (ThermoFisher Scientific, Waltham, Massachusetts, USA) was added (10 µg/mL in sterile PBS) and cell were incubated for 15 min. Cover slips were then washed once in PBS, dried, protected with Fluoroshield Antifade Mounting Medium (Abcam, Cambridge, UK) and placed on microscopy slides. Images were recorded using a Leica TCS SP8 X confocal laser scanning microscope (Leica, Munich, Germany) equipped with a supercontinuum excitation laser and tunable spectral detector. Z-stack images were acquired in order to confirm nanoparticle were localized within cells and not attached to glass surface or outer layer of cell membrane. Separately acquired channels were combined in ImageJ editor (University of Wisconsin-Madison, Madison, WI, USA).

### 2.6. Comet Assay 

DNA damage in mNSCs was measured by the use of an alkaline version of the Comet assay. Isolated hPBMCs were seeded at the concentration of 10^6^ cells/mL in 24-well plastic plates in RPMI medium (Gibco, Schwalbach, Germany) without the fetal bovine serum in CO_2_ incubator (Heraeus, Hanau, Germany) (95% humidity, 5% of CO_2_ and 37 °C) and treated with 0.2 and 1 mg/L AgNPs for 3 h. These two concentration were chosen due to previously performed comet assay experiments in which higher concentration demonstrated total genotoxic effect and cell death in Comet assay. Control cells were incubated only with RPMI medium with the same amount of UPW that corresponds to the amount for AgNPs treatment. During incubation, monocytes were attached to the bottom and lymphocytes were dispersed in supernatant with RPMI. After the treatment, lymphocytes were washed with centrifugation from exceeding AgNPs and RPMI medium was added in order to get concentration of 10^4^ cells/mL.

On each dried microscopic slide previously precoated with 200 µL of 1% normal-melting agarose (Sigma Aldrich, Darmstadt, Germany) a cell suspension was added made of 10 µL cell suspension mixed with 100 µL of 0.5% low-melting agarose (Sigma Aldrich, Darmstadt, Germany). Slides were solidified on 4 °C for 10 min and then transferred into fresh cold lysis solution and left there for 24 h. Afterwards, slides were placed in a freshly prepared denaturation and electrophoresis buffer (pH 13) on 4 °C for 20 min. Slides were transferred into electrophoresis tank with new cold electrophoresis buffer and horizontal electrophoresis was performed at 25 V and 300 mA for 20 min. Slides were neutralized 3 times for 5 min each time with Tris-buffer (pH 7.5) and stained with ethidium bromide solution (20 mg/L) for 10 min and were examined the same day. Experiments were performed in duplicate. A minimum of 50 comets per slide (100 comets per sample) were scored. Slides were analyzed with fluorescent microscope (Zeiss, Jena, Germany) attached to a black-and-white CCD camera and image analysis system (Comet assay IV; Perceptive instruments Ltd., Instem, UK). The parameters used for the quantification of DNA damage were tail length of the comet (TL, µm) and tail intensity (TI, % DNA in comet tail). The results were presented as the mean ± SD (standard deviation of the mean), standard error (SE), median and range of measured parameters.

### 2.7. Statistical Analysis 

In order to determine cell death, apoptosis induction, AgNPs uptake, oxidative stress response and mitochondrial membrane damages, there were six experiments performed for each parameter. All measurements were repeated three times for each experiment. All results are presented as the mean of six experiments including standard deviation (SD). Differences between treatments for the different measured variables were tested using the *t*-test, Mann–Whitney U-test, one-way analysis of variance (ANOVA) with post-hoc Scheffé test and Pearson’s χ^2^ test. In other cases, the differences against negative controls were tested using the Dunnett test, or Kruskal–Wallis ANOVA by Ranks test (nonparametric test used when assumptions of homogeneity of variances were not reachable). The minimal significance level for all analyzed parameters was *p* < 0.05. 

Comet assay results were analyzed with one-way break down ANOVA with Scheffe post hoc modification and results from each person in duplicate were compared. Since the results did not differ for each individual, 100 comets were analyzed together for each individual, and in group analysis, 600 comets were analyzed for each concentration and type of coated nanoparticles. 

All statistical analyses were performed using Statistica 10.0 (Statsoft, Inc., Tulsa, OK, USA).

## 3. Results and Discussion

### 3.1. Characteristics and Stability of AgNPs

**Figure 1:**
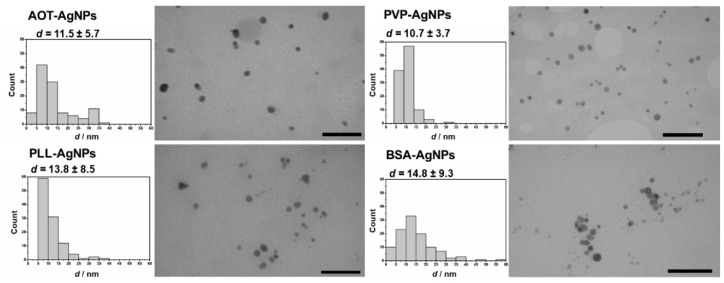
Transmission electron micrographs (TEM) show shape of different AgNPs coated with AOT, PVP, PLL and BSA dispersed in ultrapure water. Scale bars are in 100 nm. Size histograms and primary sizes (*d*, nm) of each AgNPs were obtained from the cross-sectional area of the particles as described in Supplementary Information.

For the treatment of hPBMC, well characterized AgNPs were used that were freshly synthesized before cell experiments. Synthetic procedures for these AgNPs were developed and validated in our laboratory as reported previously [24,25]. Characterization and stability evaluation performed by DLS, ELS, TEM and GFAAS (Figure 1 and Table 1) confirmed that AgNPs were of desired colloidal stability and properties, i.e., spherical in shape and 10 nm sized. Primary size of all AgNPs ranged between 10.7 and 14.3 nm, while DLS measurements showed bimodal volume-weighted size distribution (Table 1). The ELS data confirmed positive and negative ζ potential for PLL-AgNPs and AOT-AgNPs (Table 1) originating from positively charged PLL and negatively charged AOT. For PVP-AgNPs, slightly negative ζ potentials was observed as a result of the BH_4_^-^ anions attachment to the surface of PVPAgNPs during synthesis. Overall negative charge of BSA molecule contributed to negative ζ potential of BSA-AgNPs.

Colloidal stability and behavior of these AgNPs in different biological media were subject of our previous studies and reported elsewhere [24,25,26]. The presence of proteins in the media provided excellent colloidal stability regardless of the chemical composition, surface structure and surface charge of AgNPs due to the protein corona formation on the nanosurface [27]. As hPBMC isolates were dispersed in PBS before treatment, stability of AgNPs was inspected in this medium. Due to high ionic strength of PBS, destabilization of AgNPs were expected. Contrary to the pure PBS where AgNPs were agglomerated (Table 2), we did not observe visually precipitation of any AgNPs for PBS containing hPBMC. Although it was not possible to fully examine agglomeration behavior of AgNPs by DLS due to the presence of hPBMC, quantification of proteins revealed total protein content of 24 mg/L in PBS + hPBMC media excluding protein content of hPBMC. It is well known that protein corona forms on particle surface already within first minutes after dispersion of NPs in biological fluid providing colloidal stability regardless of NPs phyisico-chemical properties [24,28]. For all AgNPs types enclosed by this study, we already proved such stabilization [24]. Indeed, formation of protein corona in PBS + hPBMC medium was tested by incubating each AgNPs in this medium for 1 h using concentration of 25 mg Ag/L. However, to exclude effect of hPBMC themselves, cells were first removed from PBS + hPBMC media by centrifugation and AgNPs were incubated in supernatant that contained 24 mg proteins/L. Results revealed that more than 80% of present proteins attached to the AgNPs surface (Table 2). There were no significant differences in the amount of proteins between different AgNPs. According to Tenzer et al. [29], the amount of adsorbed proteins would not change significantly after longer incubation.

Dissolution of AgNPs significantly decreased in both PBS and PBS + hPBMC media (Table 2). In the PBS, lower amount of free Ag^+^ ions may be explained by the attachment of ionic Ag and Ag complex ions on the surface of AgNP agglomerates [15,16]. As expected, presence of proteins in PBS-hPBMC and protein corona formation on particle surface prevented significantly the dissolution of AgNPs in agreement with our previously published data [17,18,24]. Amount of free Ag^+^ ions in this medium ranged between 0.1 and 0.4% (*v*/*v*) (Table 2). These results were important for later distinction between nanoparticle- and Ag ions-related effects on hPBMC.

### 3.2. Cytotoxicity Effects and Cellular Uptake of AgNPs 

**Figure 2:**
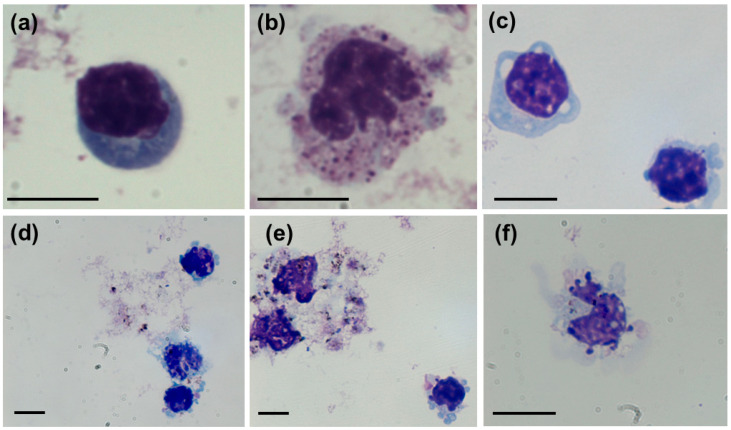
Light microscopy images of control, untreated (**a**) lymphocyte and (**b**) monocyte. Treatment of AgNPs coated with AOT, PVP, PLL and BSA induced morphological alterations of human peripheral blood mononuclear cells (hPBMC). (**c**) Vacuolization and apoptotic bodies in lymphocytes treated with AOT-AgNPs. (**d**) Alterations in lymphocytes and monocytes treated with PVP-AgNPs. (**e**) Necrotic cells (in the upper left corner) and apoptotic bodies in lymphocytes (down right corner) after treatment with PLL-AgNPs. (**f**) Intracytoplasmic vacuoles, enlarged multi-lobed nuclei and ruffled surface of monocyte treated with BSA-AgNPs. Scale bars are in 10 μm.

**Figure 3:**
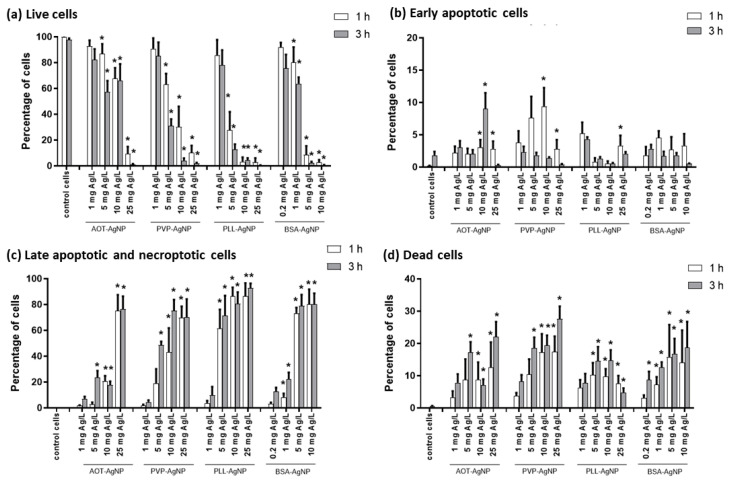
The effect of AOT-AgNPs, PVP-AgNPs, PLL-AgNPs, and BSA-AgNPs on the % of (**a**) live cells, (**b**) early apoptotic cells, (**c**) late apoptotic, necroptotic and secondary necrotic cells, and (**d**) dead cells, as determined by flow cytometry after Annexin V/PI staining. The hPBMC were exposed to AgNPs for 1 h (white columns) and 3 h (grey columns). Controls were untreated cells. The results are expressed as percentage of controls and given as mean values obtained from six independent experiments including SD as error bars. Values marked with asterisk (*) differ significantly from the negative control (*p* < 0.05).

Toxicity effects of differently coated AgNPs were evaluated at fixed incubation times of one and three hours owing to sensitivity of freshly isolated hPBMC. Visualization of cells by light microscopy indicated AgNPs-induced cytomorphological alterations (Figure 2). Treated cells were characterized by the presence of intracytoplasmic vacuoles, enlarged multi-lobed nuclei, ruffled cell surface, apoptotic bodies and condensed chromatin that are clear sign of mechanical stress and apoptosis (Figure 3). Similar morphological changes of hPBMC treated with AgNPs were reported by Zhornik et al. [30] and Joksić et al. [31].

Flow cytometry experiments using FITC Annexin V Apoptosis Detection Kit I allowed us to evaluate ability of different AgNPs to induce apoptosis and cell death in hPBMC. All four types of tested AgNPs induced cell death and apoptosis in a dose- and time-response (Figure 3). Significant death of hPBMC was observed already after 1 h for AgNPs doses up to 10 mg Ag/L, while 3 h incubation decreased “safe” dose of AgNPs.

Staining of cells with Annexin V clearly demonstrated ability of all AgNPs to induce apoptosis of hPBMC. However, toxic potential was dependent on the type of coating agent used for AgNPs stabilization. Highly negative AOT-AgNPs induced significant increase in the apoptotic rate (3% and 20.5% cells in early and late apoptosis, respectively) was observed after 1 h for concentration of 10 mg Ag/L, while lower concentration of 5 mg Ag/L induced significant apoptosis (23.5% cells) and cell death (17.2% of cells) after 3 h. Particles stabilized with neutral polymer PVP showed similar behaviour as AOT-AgNPs, but induced apoptosis and cell death in much larger cell population. Thus, 1 h treatment with 10 mg Ag/L of PVP-AgNPs led to 43.2% hPBMC in late apoptosis and 17.2% dead cells, while significant effects after longer treatment (3 h) were observed for 5 mg Ag/L PVP-AgNPs (48.6% hPBMC in late apoptosis and 18.6% dead cells). Positively charged PLL-AgNPs exhibited much higher toxicity. Concentration of 5 mg Ag/L induced already after 1 h treatment apoptosis in 61.5% hPBMC and death of 10.2% cells (Figure 2). The highest toxicity was observed for BSA-AgNPs where doses of 25 mg Ag/L completely destroyed hPBMC, while significant apoptosis and cell death occurred at dose of 1 mg Ag/L of BSA-AgNPs (Figure 2). Any effect of coating agents (PLL, PVP and AOT) used for AgNPs stabilization was also excluded by additional control experiments performed at concentrations that correspond to the amount of coating agents in dispersion of 25 mg Ag/L AgNPs (Figure S6 in the Supporting Materials). Results showed that PVP did not affect significantly % of live, early apoptotic, late apoptotic and dead hPBMC, while AOT and PLL induced early apoptosis in 8.8 and 21.0% cells, respectively.

Similar results on AgNPs ability to induce apoptosis was previously reported for citrate-coated AgNPs on cetacean polymorphonuclear cells and cetacean PBMC [32], for 70 nm sized PVP-AgNPs in human monocytic cell line THP-1 [10], and for hPBMC treated with poly(vinyl alcohol)-coated AgNPs [33] or non-coated ultra-small Ag nanoclusters [34]. In all these studies, concentration range of tested Ag-based nanomaterials were similar to concentration levels used in this study, i.e., 0.1 to 50 mg Ag/L (see Table S1 in the Supporting Materials). Despite huge amount of scientific data on AgNPs toxicity effects, comprehensive and systematic data on biological response of hPBMC to AgNPs are scarce. We compared our results also with some reports on viability, proliferation, inflammatory and oxidative stress response of hPBMC treated with AgNPs [15,19,20,21,22,23,35,36]. Most of them tested also effects of free Ag^+^ ions that may be released in biological media from the AgNPs surface. This issue has been and it is still being discussed in scientific community. For tested AOT-, PVP-, PLL- and BSA-coated AgNPs, the highest amount of released Ag^+^ ions was detected to be 1.1% in ultrapure water in the case of PLL-AgNPs, while this amount decreased to 0.4% in PBS + hPBMC media (see Table 1 and Table 2). If we consider the highest possible ionic Ag fraction that may occur, AOT-AgNPs and PVP-AgNPs may release 0.004, 0.02, 0.04 and 0.1 mg Ag^+^/L, PLL-AgNPs may release 0.011, 0.055, 0.11 and 0.275 mg Ag^+^/L and BSA-AgNPs may release 0.008, 0.04, 0.08 and 0.2 mg Ag^+^/L at AgNPs concentrations of 1, 5, 10 and 25 mg Ag/L, respectively. Control experiments with Ag^+^ ions (in the form of AgNO_3_) showed that ionic Ag did not significantly affect hPBMC up to the concentration of 1 mg Ag^+^/L (Figure S6 in the Supporting Materials). Therefore, we may conclude that dissolution behavior of AgNPs in test media did not interfere with cytotoxicity effects of nanoparticles (Figure 3). However, intracellular decomposition and intracellular release of Ag^+^ ions should be considered as well. This may lead to the toxicity mechanism named as “lysosome-enhanced trojan horse“ (LETH) effect and suggested by Sabella et al. [37].

**Figure 4:**
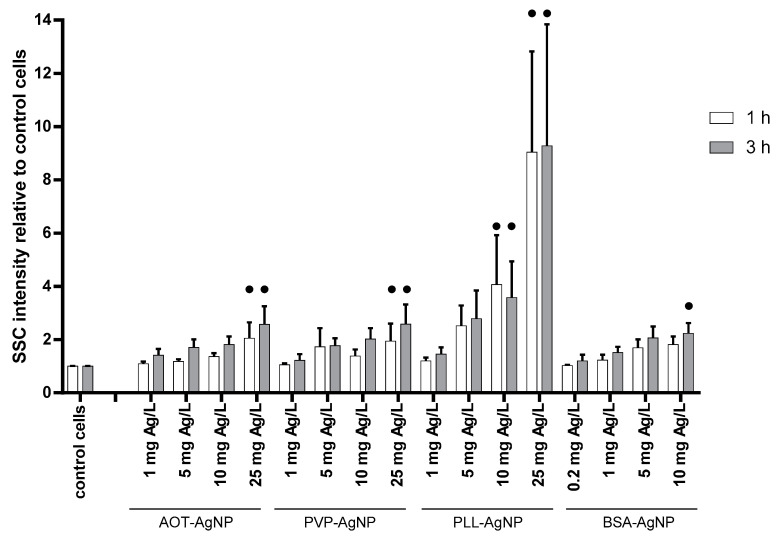
Uptake of AOT-, PVP-, PLL- and BSA-coated AgNPs by hPBMC cells analysed by the flow cytometry. Cells were exposed to different concentrations of AgNPs, given in mg Ag/L, for 1 (white columns) and 3 h (grey columns). Control cells were cultivated in AgNPs-free exposure media (negative controls). The percentage of relative uptake, calculated as percentages of the increase of the side scattered light of the laser beam (SSC) relative to control cells, is expressed as the mean of six independent experiments including SD as error bars. Significant differences between treated and control cells are marked with dots (•) at *p* > 0.05.

Any intracellular AgNPs-related event is not possible without internalization of particles by hPBMC, which was evaluated by combining flow cytometry and confocal microscopy. Flow cytometry experiments provided data on the SSC intensity originating from the reflection of light by intracellular structures [38], which enabled analysis of AgNPs uptake as internalized particles change. Observed increase in the SSC signals of AgNPs-treated hPBMC were dose-dependent for all tested particles (Figure 4). It should be highlighted that analysis of SSC intensity was performed only for live hPBMC. There were no significant differences between uptake after 1 and 3 h. The highest SSC signals were observed for hPBMC treated with PLL-AgNPs, which may be explained by promotion of particles uptake due to the electrostatic interaction of positively particles with negatively charged cell surface (Figure 4). The uptake for PVP- and AOT-coated AgNPs was similar (Figure 4), as we already observed for human hepatoma cells [39], probably as a result of protein corona formed on the AgNPs surface that favored interaction with cell membrane and active internalization of particles despite the unfavorable electrostatic interaction [29,40]. Results obtained for BSA-AgNPs showed the same dose-response in NPs uptake, that were significantly increased compared to control already at concentration of 10 mg Ag/L (Figure 4). Dose–response uptake of AgNPs by hPBMC was also reported by other authors. Thus, Greulich et al. evidenced cell uptake of 70 nm sized PVP-AgNPs by combining flow cytometry and scanning electron microscopy [38], while Vergallo et al. quantified internalized β-D-glucose-coated AgNPs by GF-AAS [41].

**Figure 5:**
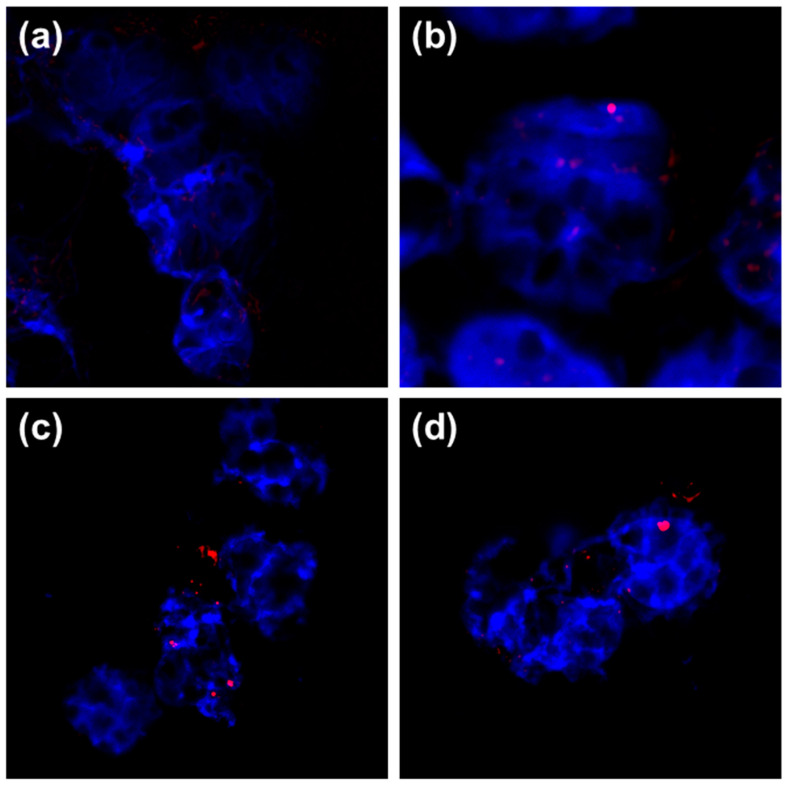
Visualization of AgNPs uptake by hPMBC performed by contrast reflection mode of CLSM. Untreated control cells (**a**) were compared to cells treated with AOT-AgNP (**b**), PVP-AgNPs (**c**) and PLL-AgNPs (**d**) for 3 h and at concentration of 1 mg Ag/L. The images show maximum intensity Z-projections of cells. Nucleic acid staining was performed using Hoechst 33,258 fluorescent dye (blue) to stain nuclei, while CLSM reflectance signals (red) are visible as bright red signals (**b**–**d**) indicating the presence of AgNPs.

Flow cytometry experiments did not allow to discriminate AgNPs adsorption and binding on the cell surface from their internalization. Visualization using the reflection contrast mode of CLSM directly evidenced that hPBMC internalized all types of AgNPs (Figure 5). Although the pore size of lymphocyte membrane (ca. 4 × 2.5 nm) is smaller than diameter of tested AgNPs, internalization may occur by active endocytic pathways or as a consequence of cell deformity, cell membrane damage and vacuolization as reported by Li et al. for cetacean PBMC [32]. In all treated cells, accumulated AgNPs were clearly visible intracellularly and within the nuclei (Figure 5b–d). Ability for metallic NPs to penetrate nuclei of lymphocytes were described earlier for paramagnetic iron oxide NPs [42] and gold NPs [43].

**Figure 6:**
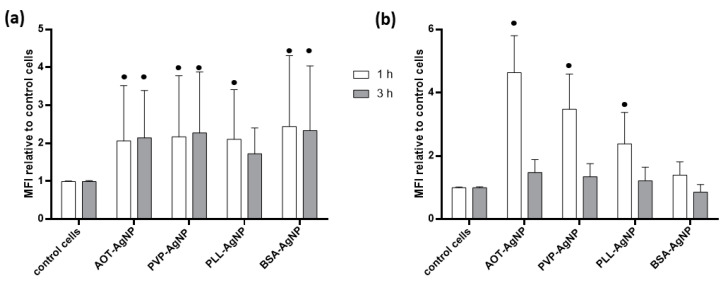
Oxidative stress response of hPBMC treated with different AgNPs for 1 (white columns) and 3 h (grey columns) at concentration of 1 mg Ag/L. ROS levels were determined using (**a**) DHE and (**b**) DCFH-DA assays. Data are given as ratio of geometrical MFI of treated relative to control, non-treated cells. All results are presented as the mean of 6 experiments including SD represented as error bars. Significant changes between treatments and control cells are denoted by dots (●) (*p* < 0.05).

As a consequence of AgNPs internalization, initiation of LETH mechanism may be expected leading to excessive production of reactive oxygen species (ROS) and oxidative stress response as already recognized for many cell types treated with metallic NPs [37] and evidenced also for hPBMC [33,34,37,38,41,44,45]. Oxidative stress response of hPBMC to treatment with different AgNPs was evaluated for dose of 1 mg Ag/L that did not induce any significant changes in the number of apoptotic or dead cells. Both DHE and DCFH-DA assays evidenced that all tested AgNPs significantly increased ROS level in hPBMC (Figure 6). Induction of oxidative stress in hPBMC treated with such small dose of AgNPs was also reported by other authors as presented in Table S1 of Supporting Materials [33,34,38,44,45].

A key role of ROS in toxicity of AgNPs was established more than a decade ago [46]. Thus, oxidation of AgNPs may lead to the production of superoxide radical via Fenton-like mechanism [46] and superoxide radical, that can be detected by DHE assay is precursor of hydroxyl-, perhydroxyl- and peroxyl-radicals [47], usually measured by DCFH-DA assay. Production of superoxide radicals was pronounced similarly for all AgNPs types irrespective of incubation time, while peroxy radicals were detected only in hPBMC treated for 1 h with AOT-, PVP- and PLL-coated AgNPs.

**Figure 7:**
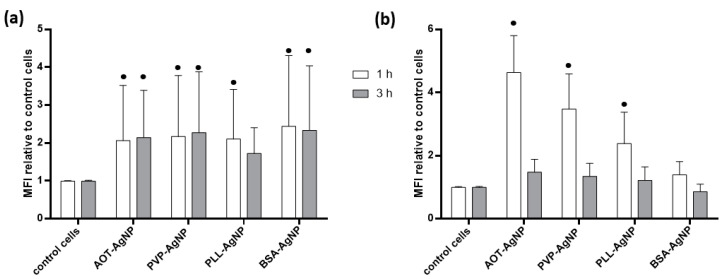
Effect of different AgNPs on mitochondrial membrane potential of hPBMC determined by DiOC_6_ staining after 1 (white columns) and 3 h (grey columns) of exposure. Data are given as ratio of geometrical MFI of treated relative to control, nontreated cells. All results are presented as the mean of 6 experiments including SD represented as error bars. Significant differences between treatments and control cells are denoted by dots (●) (*p* < 0.05).

It may be concluded that AgNPs-observed cytotoxicity was ROS-dependent. Oxidative stress may disrupt mitochondrial function and intracellular ROS generation has been postulated as an important mitochondrial mechanism of AgNPs cytotoxicity [48]. In this study, the assessment of changes in the mitochondrial membrane potential evidenced significant decrease in DiOC_6_ fluorescence signals of treated vs. control hPBMC for both incubation time (Figure 7). 

Such decrease is indicative for mitochondrial membrane depolarization that leads to opening of the mitochondrial permeability pores and release of DiOC_6_ dye from mitochondria. Thus, all tested AgNPs induced depolarization of mitochondrial membrane by similar extent (Figure 7). The MMP and ROS results corroborate well with observed apoptosis and necrosis of hPBMC treated with different AgNPs. Damaged mitochondria produce more ROS, especially superoxide radicals, while excessive ROS induce a rapid depolarization of mitochondrial membrane leading to an impairment of oxidative phosphorylation [49]. Thus, observed increase in ROS level in hPBMC following the treatment with AgNPs may be resultant of both AgNPs oxidation and damaged mitochondria. The major ROS action inside the mitochondria is mitochondrial permeability transition leading to immediate dissipation of the mitochondrial membrane potential leading to the opening of the mitochondrial permeability transition pores and subsequent release of apoptogenic factors [50]. Observed apoptosis in hPBMC treated for 1 h with AgNPs (Figure 3) complies to this mechanism. Progression from early to late apoptosis and from late apoptosis to cell death after longer incubation (i.e., 3 h) implies that AgNPs drive apoptosis as main cell death mechanism in hPBMC.

### 3.3. Genotoxicity of AgNPs

Excessive amount of ROS in the cells may initiate many destructive mechanisms including degradation and fragmentation of DNA. DNA damage was evaluated by Comet assay in AgNPs-treated lymphocytes. Two parameters of DNA damage were measured, TL and TI. The results are shown as mean and standard deviation in Table 3, while raw data are given in Table S1 of Supporting Materials. 

For each type of AgNPs, two doses were tested, 0.2 and 1 mg Ag/L. Depending on the dose, all AgNPs induced significant increase in the TL or TI or both. Lower dose of PVP- and BSA-coated AgNPs (0.2 mg Ag/L) did not induce significant change of TL, while TI did not change after treatment with lower dose of PLL- and PVP-AgNPs. Significant increase of TL indicated that fragmentation of the DNA started, but only the TI parameter reliably indicated the percentage of damaged DNA in tails. Similar genotoxic effects were also evidenced for hPBMC treated by small sized non-coated Ag nanoclusters [34] and non-coated AgNPs [31]. 

For the TL, values for mean, median and ranges were similar for treatment with higher dose of AgNPs irrespective of the particle type, while the highest %DNA in tail was found for lymphocytes treated with higher concentration (1 mg Ag/L) of BSA-AgNPs. As genotoxic effect was observed after only 3 h incubation, we assumed that it may be induced not only by ROS production and apoptosis induction, but also by direct interaction of AgNPs with DNA owing to the fact that AgNPs were found also in nuclei (Figure 5). Considering TI mean values, genotoxic potential of AgNPs was dependent on the surface coating and followed the order PVP-AgNPs < PLL-AgNPs < AOT-AgNPs < BSA-AgNPs.

## 4. Conclusions

This study provides for the first time comprehensive and systematic assessment how differently functionalized AgNPs affect human PBMC. All AgNPs types induced apoptosis in a dose-response manner after short time, i.e., already after 1 h. Despite their colloidal stability in biological media, their efficient internalization by hPBMC enabled intracellular dissolution and triggering of LETH mechanism. Such mechanism includes AgNP oxidation and release of ionic Ag leading to the ROS production and subsequent apoptotic cell death. Obtained results clearly indicate immunotoxicity of AgNPs that should be taken into account in future safety assessment of nanosilver-based medical products. Particular attention should be placed on blood-contacting materials that require careful evaluation of hemocompatibility.

## Figures and Tables

**Table 1:** Physico-chemical characteristics of different AgNPs. Coating agents used for stabilization were AOT, PVP, PLL and BSA. Hydrodynamic diameter (*d*_H_, nm) values were obtained from size distributions by volume using DLS and ζ potential values (mV) were measured by ELS method. The % of free Ag^+^ ions was measured by GFAAS in filtrates obtained by centrifugal ultrafiltration of AgNPs dispersions. All data were obtained for AgNPs dispersed in ultrapure water at concentrations of 10 mg Ag/L and at 25 °C.

NPs Type	d_H_ (nm)	% Mean Volume	ζ Potential (mV)	% Ag^+^
**AOT-AgNPs**	14.9 ± 4.5	92.2	−39.8 ± 3.8	0.4
	28.4 ± 6.7	7.8		
**PLL-AgNPs**	5.8 ± 1.6	94.8	−19.3 ± 2.6	0.4
	29.8 ± 6.1	5.2		
**PVP-AgNPs**	8.8 ± 0.9	95.7	40.6 ± 1.5	1.1
	52.6 ± 16.3	4.3		
**BSA-AgNPs**	14.1 ± 3.3	86.3	−10.6 ± 3.2	0.8
	58.4 ± 18.1	13.7		

**Table 2:** Stability of AgNPs coated with AOT, PVP, PLL and BSA in PBS and PBS with addition of hPBMC. Hydrodynamic diameter (*d*_H_, nm) values obtained from size distributions by volume and ζ potential values (mV) were measured 1 h after dispersion of AgNPs in PBS, while % of free Ag^+^ ions was measured by GFAAS in filtrates obtained by centrifugal ultrafiltration of AgNPs dispersed in PBS and PBS + hPBMC media for 1 h at concentrations of 10 mg Ag/L and at 25 °C. Quantitative analysis of protein corona formed on the surface AgNPs after 1 h incubation in PBS + hPBMC media (excluding cells) at 37 °C is given as mg proteins/NP.

NPs Type	PBS			PBS + hPBMC	
	*d*_H_, nm (% Mean Volume)	*ζ* Potential (mV)	% Ag^+^	μg Proteins/mg Ag	% Ag^+^
**AOT-AgNPs**	32.4 ± 7.9 (15%) 328.3 ± 28.7 (85%)	−19.1 ± 4.2	0.3	796 ± 58	0.1
**PVP-AgNPs**	12.7 ± 8.1 (8%) 408.9 ± 67.7 (92%)	−13.7 ± 3.3	0.1	714 ± 43	0.3
**PLL-AgNPs**	12.8 ± 6.3 (18%) 512.5 ± 91.4 (82%)	−14.1 ± 2.5	0.8	803 ± 62	0.4
**BSA-AgNPs**	11.8 ± 4.6 (2%) 198.7 ± 56.4 (98%)	−15.4 ± 2.8	0.7	842 ± 74	0.2

**Table 3:** Results of Comet assay in lymphocytes treated with AOT-, PVP-, PLL- and BSA-coated AgNPs for 3 h. Obtained parameters, tail length (TL) and tail intensity (TI), are expressed as mean values including SD, medians and ranges. Results are obtained from 6 independent experiments. Statistically significant difference are denoted with asterisk (*) for the mean TI and TL values of treated compared to non-treated control cells at the *p* < 0.05.

AgNPs Type	Dose, mg Ag/L	TL (µm)			TI (%DNA in Tail)		
		Mean ± SD	Median	Range	Mean ± SD	Median	Range
Control	0	16.80 ± 4.27	16.25	8.75–31.25	0.30 ± 0.61	0.07	0.00–8.51
AOT-AgNPs	0.2	19.07 ± 5.00 *	17.29	10.83–36.67	0.49 ± 0.55 *	0.31	0.00–3.03
	1	18.45 ± 4.19 *	17.50	11.67–40.83	0.70 ± 0.94 *	0.38	0.00–5.84
BSA-AgNPs	0.2	17.55 ± 4.01	16.67	10.83–34.17	0.51 ± 0.58 *	0.30	0.00–2.97
	1	19.19 ± 4.74 *	17.92	10.42–34.58	0.88 ± 0.91 *	0.61	0.00–6.33
PLL-AgNPs	0.2	18.20 ± 3.90 *	16.67	12.08–30.83	0.31 ± 0.33	0.20	0.00–1.91
	1	18.70 ± 4.54 *	17.50	12.08–34.58	0.61 ± 0.73 *	0.39	0.00–4.50
PVP-AgNPs	0.2	17.06 ± 3.66	16.25	10.00–30.00	0.26 ± 0.35	0.09	0.00–2.39
	1	18.40 ± 4.50 *	17.08	12.08–31.17	0.49 ± 0.58 *	0.27	0.00–3.32

## Supplementary Materials

The following are available online at https://www.mdpi.com/2079-4991/10/7/1390/s1: Detail on synthesis and characterization of silver nanoparticles. Table S1: Published results on AgNPs effects on PBMC. Figure S1: Gating strategy flow cytometry evaluation of AgNPs uptake and cytotoxicity in hPBMC. Figure S2: FCM dot-plot analysis of apoptotic/necrotic cells after treatment with different AgNPs at concentration of 5 mg Ag/L for 1 h, using FMO controls. Figure S3: FCM histogram analysis of ROS production in cells stained with DCFH-DA. Figure S4: FCM histogram analysis of changes in mitochondrial membrane potential in cells stained with DiOC6. Figure S5: FCM histogram analysis of ROS production in cells stained with DHE. Figure S6: The effect of ionic silver, AOT, PVP and PLL on the % of live (white columns), early apoptotic (light grey columns), late apoptotic (dark grey columns) and dead (stripped columns) hPBMC after 3 h exposure.
