# Supplementary material for: Surface Stabilization Affects Toxicity of Silver Nanoparticles in Human Peripheral Blood Mononuclear Cells
Source: Nanomaterials (Basel). 2020 Jul 17;10(7):1390. doi: 10.3390/nano10071390 (PMC7407574; doi:10.3390/nano10071390)
Supplement: Supplementary file 1 [file nanomaterials-10-01390-s001.pdf]

Synthesis and characterization of silver nanoparticles

Silver nanoparticles (AgNPs) were synthesized by reduction of 2.3 mM AgNO3 with 4 mM NaBH4 was employed in the presence of AOT, PVP and PLL as coating agents at finally concentrations of 500, 75 and 20 mM, respectively, at room temperature. The BSA-coated AgNPs were prepared by mixing AgNO3 and NaBH4 at the molar ratio of 1:1 (at 10 mM) in the presence of 13.50 μmol BSA. The reaction was allowed to proceed for 1 h. After the synthesis, all AgNPs colloids were washed with ultrapure water (UPW) by centrifugation at 11 000 × g for 20 min. After decanting the supernatant, the residue was filtered through Amicon® Ultra centrifugal filters (Merck Millipore Ltd., Tullagreen, Carrigtwohill Co., Cok, Ireland) with the pore size of 3 kDa to remove unwanted fractions of free ionic Ag, unadsorbed coating agents or chemicals used or generated during synthesis. Purified AgNPs were then suspended in ultrapure water and kept at 4 °C in the dark.

The formation of nano-sized silver particles was verified by the presence of a Surface Plasmon Resonance (SPR) peak measured using an UV-Vis spectrophotometer (CARY 300, Varian Inc., Australia). Concentration of AgNPs was determined as mg Ag/L by measuring total Ag concentrations in AgNPs stock solutions using the graphite furnace atomic absorption spectrometer (GFAAS) (Perkin Elmer AAnalyst 600, Perkin Elmer, Shelton, USA) with Zeeman background correction. A standard solution of Ag (1000 mg Ag/L in 5% HNO3) from Merck (Darmstadt, Germany) was used for calibration.

Careful characterization of each AgNP type was conducted in ultrapure water at the concentration of 10 mg Ag/L. The size and charge of AgNPs were measured at 25 °C by dynamic (DLS) and electrophoretic light scattering (ELS) at 173° using Zetasizer Nano ZS (Malvern, UK) equipped with a green laser (532 nm). To avoid overestimations arising from the scattering of larger particles, the hydrodynamic diameter (d_H) was obtained as a value at peak maximum of size volume distribution function, reported as an average of 10 measurements. The charge of the NPs was characterized by zeta potential values, expressed as average value of 5 measurement and calculated from the measured electrophoretic mobility by means of the Henry equation using the Smoluchowski approximation. The DLS and ELS data were processed by the Zetasizer software 6.32 (Malvern Instruments). In addition, particles were visualized using a transmission electron microscope (TEM, Zeiss 902A) operated in a bright field mode at an acceleration voltage of 80 kV. Images were recorded with Canon PowerShot S50 camera attached to the microscope. TEM samples were prepared by depositing a drop of the particle suspension on a Formvar® coated copper grid and air-drying at room temperature. TEM images were also used for measurement of primary size (d) of AgNPs. Size was determined from the cross-sectional area of the particles which was converted to an equivalent spherical diameter by using ImageJ software. Primary particles were distinguished from AgNPs aggregates by tracing it manually. Altogether at least 110 particles per particle type were measured.

Released free Ag ions fraction in each AgNPs was determined by ultrafiltration across a membrane of 3 kDa Amicon-4 Ultra centrifugal filter units (Merck Millipore, Darmstadt, Germany). Obtained filtrates were analysed by the graphite furnace atomic absorption spectrometer (GFAAS) (Perkin Elmer AAnalyst 600, Perkin Elmer, Shelton, USA) with Zeeman background correction. An Ag standard solution (1000 mg/L in 5% HNO3) from Merck (Darmstadt, Germany) was used for calibration. The % of dissolution was calculated as relative ratio of Ag concentrations before and after the ultrafiltration.

Table S1. Published results on AgNPs effects on peripheral blood mononuclear cells (PBMC).

**Origin of cells**	**Type of AgNPs**	**Applied doses**	**Toxicity effect**	**Reference**
Cetecean PBMC	Commercial citrate-coated 30 nm sized AgNPs	0 – 50 mg Ag/L	Cytomorphological alterations, intracellular distribution, cytotoxicity, apoptosis induction, proliferative activity	Li, W.T.; Chang, H.W.; Yang, W.C.; et al. *Sci. Rep*. 2018, vol. 8, no. 1, pp. 1–12, doi: 10.1038/s41598-018-23737-0.
Human circulating lymphocytes	Uncoated small sized AgNPs	0 – 80 mg Ag/L	Cytomorphological alterations, genotoxicity effects	Joksić, G.; Stašić, J.; Filipović, J.; et al. *Toxicol. Lett*. 2016, vol. 247 pp. 29–34, doi: https://doi.org/10.1016/j.toxlet.2016.02.007
Human lymphocytes	Chitosan-coated AgNPs of size 5-20 nm	0 – 100 mg Ag/L	Cytomorphological and cell viability alterations, oxidative stress induction	Zhornik, E. V.; Baranova, L.A.; Drozd, E.S.; et al. *Biophys. (Russian Fed.* 2014, *vol. 59, no. 3*, pp. 380–386, doi: 10.1134/S0006350914030282
Human lymphocytes	β-D-glucose-coated 30 nm sized AgNPs	2 and 10 × 10^3 particles per cell	Cytomorphological alterations, internalization, altered cell viability, oxidative stress induction	Vergallo, C.; Panzarini, E.; Izzo, D.; et al. *AIP Conf. Proc*. 2014, vol. 1603, no. February 2015, pp. 78–85, doi: 10.1063/1.4883045
Human PBMC	PVP-coated 70 nm sized AgNPs	0 – 30 mg Ag/L	Particle internalization, altered cell viability and proliferation, cytokine release, oxidative stress induction	Greulich, C.; Diendorf, J.; Geßmann, J.; et al. *Acta Biomater*. 2011, vol. 7, no. 9, pp. 3505–3514, doi: https://doi.org/10.1016/j.actbio.2011.05.030
Mouse spleen and human lymphocytes	Citrate-coated 70 nm sized AgNPs	0 – 200 mg Ag/L	Altered cell viability, mitogen-induced proliferation, primary immunotoxicological functional alterations	Devanabanda, M.; Latheef, S.A.; Madduri, R. *J. Immunotoxicol*. 2016, vol. 13, no. 6, pp. 897–902, doi: 10.1080/1547691X.2016.1234522
Human PBMC	Citrate- and PVP-coated 20 nm sized AgNPs	0 – 80 mg Ag/L	Altered cell proliferation	Huan, H.; Lai, W.; Cui, M.; et al. *Sci. Rep*. 2016, 6, 25518. Doi: 10.1038/srep25518.
Human PBMC	Small sized uncoated Ag nanoclusters	0 – 5 mg Ag/L	Altered cell viability, oxidative stress and apoptosis induction, genotoxicity, particle internalization	Orta-García ST, Plascencia-Villa G, Ochoa-Martínez AC, et al. *J Appl Toxicol*. 2015;35(10):1189-1199. doi:10.1002/jat.3190
Human PBMC	PVA-coated small sized AgNPs	0, 1 and 50 µM	Altered cell viability, oxidative stress and apoptosis induction, genotoxicity, particle internalization	Paino IM, Zucolotto V. *Environ Toxicol Pharmacol*. 2015; 39(2): 614-621. doi:10.1016/j.etap.2014.12.012
Human PBMC	AOT-, PVP, PLL- and BSA-coated small sized AgNPs	0 – 5 mg Ag/L	Cytomorphological alterations, intracellular distribution, apoptosis and oxidative stress induction, mitoshondrial membrane damages, genotoxicity	**This study**

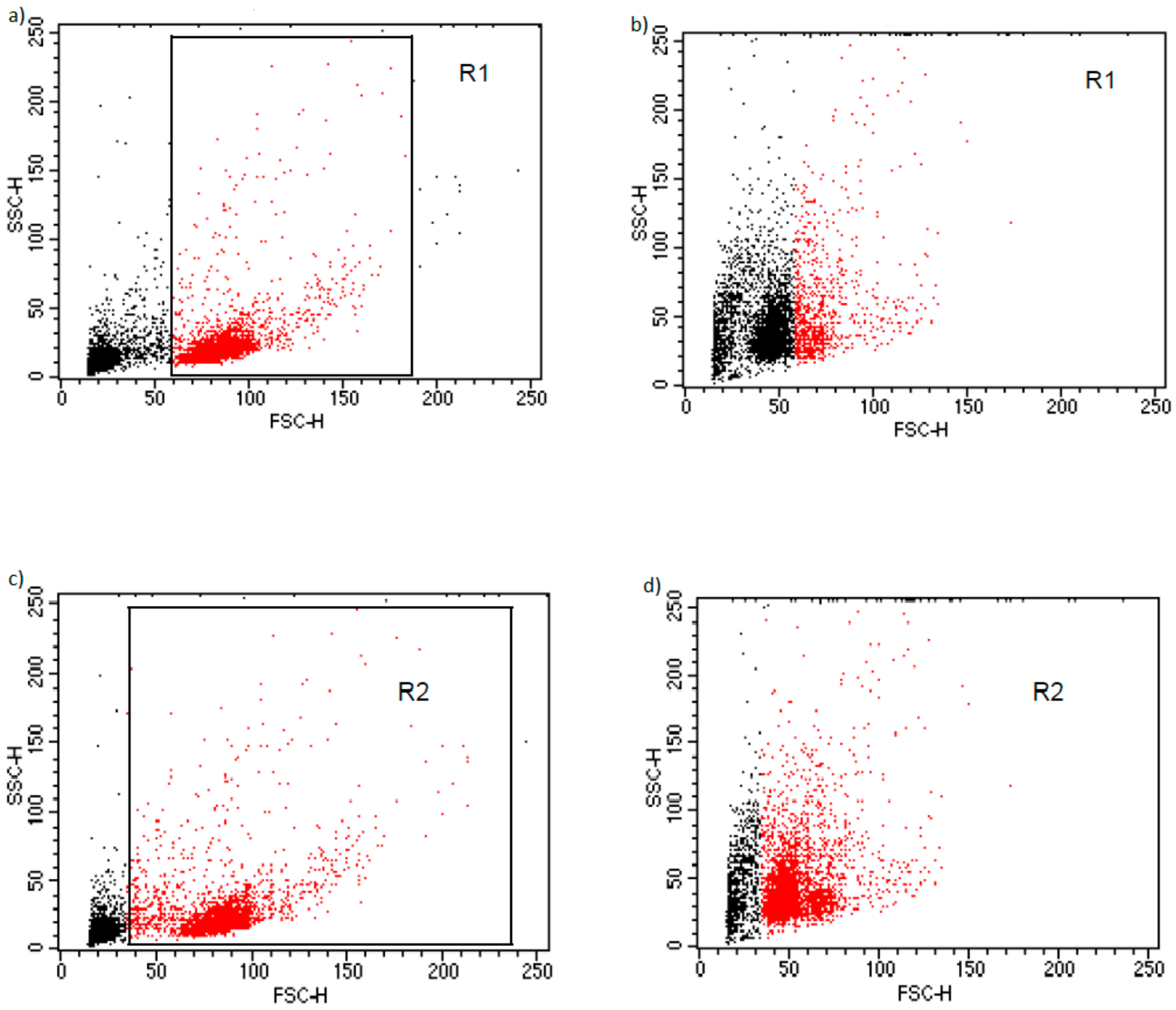

Figure S1. Gating strategy flow cytometry evaluation of AgNPs uptake and cytotoxicity in hPBMC. Uptake of AgNPs was evaluated only in living cells (R1). The gate was set on untreated cells (a) and then copied to cells treated with AgNPs (b). Cytotoxicity of AgNPs was evaluated on all hPBMC excluding cell debris (R2). The gate was set on untreated cells (c) and then copied to cells treated with AgNPs (d).

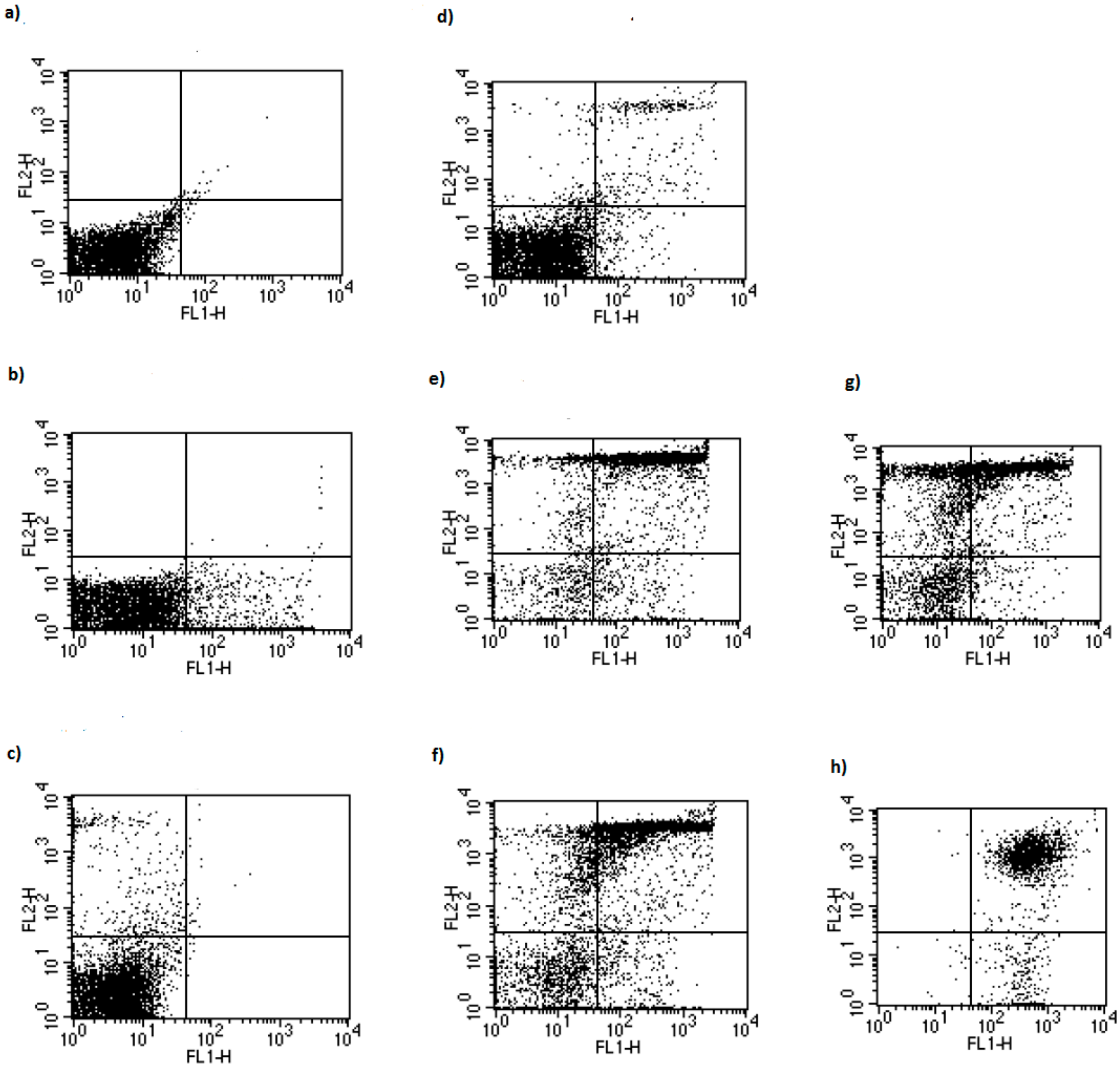

Figure S2. FCM dot-plot analysis of apoptotic/necrotic cells after treatment with different AgNPs at concentration of 5 mg Ag/L for 1 h, using FMO controls. Quadrant was first set on unstained and untreated cells (a) and then adjusted with FMO positive controls without PI staining (FL2-H) (b) and without FITC Annexin V staining (FL1-H (c). Adjusted quadrant was confirmed by double-stained positive controls (d) and copied to dot plots of treated cells with PVP-AgNPs (e), BSA-AgNPs (f), AOT-AgNPs (g) and PLL-AgNPs (h). Lower left quadrants represent live cells (FITC Annexin V-/PI-), lower right quadrants represent early apoptotic cells (FITC Annexin V+/PI-), upper right quadrants represent late apoptotic cells (FITC Annexin V+/PI+), and upper left quadrants represent necrotic cells (FITC Annexin V-/PI+).

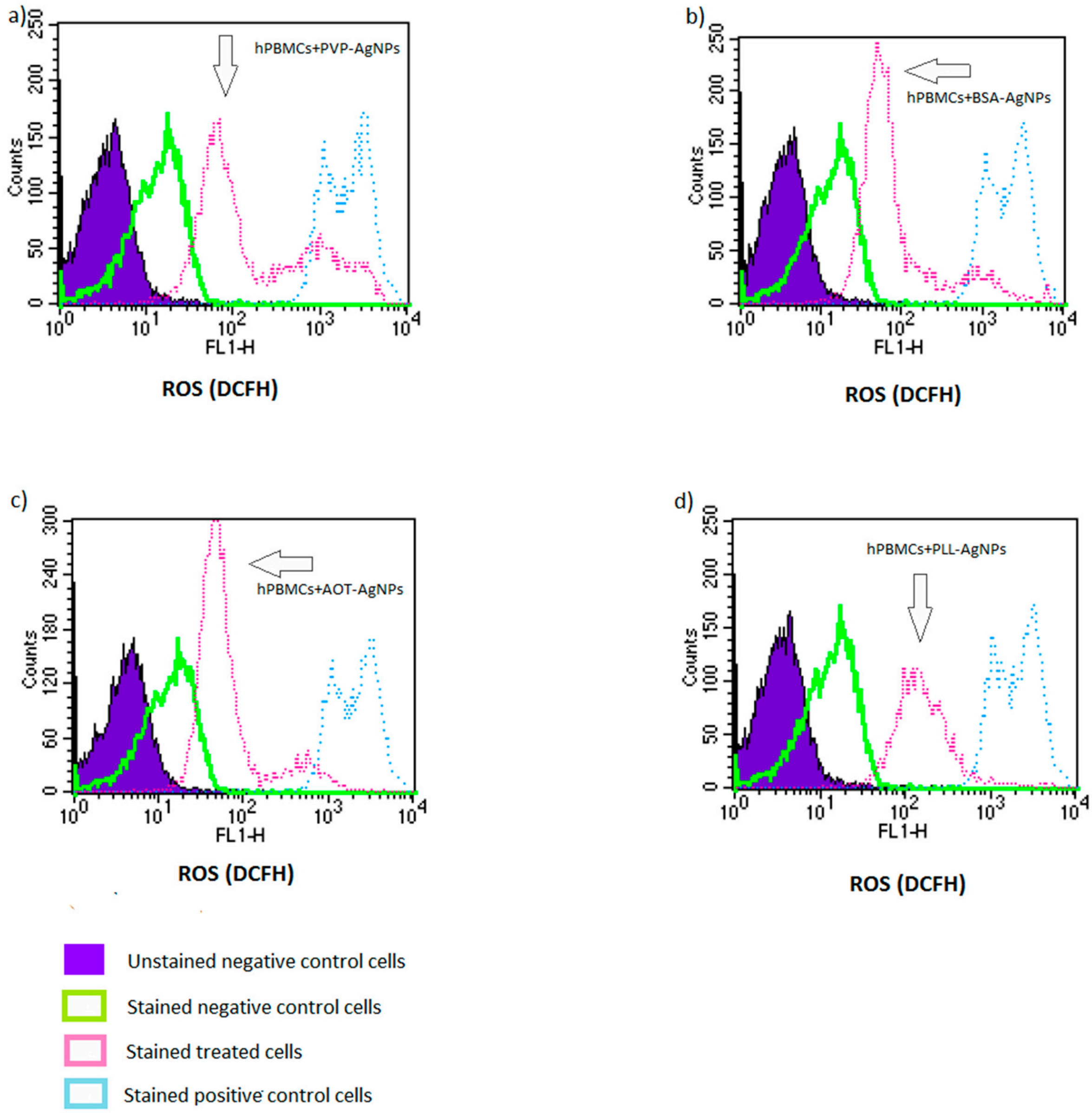

Figure S3. FCM histogram analysis of ROS production in cells stained with DCFH-DA. Cells treated with PVP-AgNPs (a), BSA-AgNPs (b), AOT-AgNPs (c) and PLL-AgNPs (d) show an increase in fluorescence intensity (FL1-H) compared to stained negative controls.

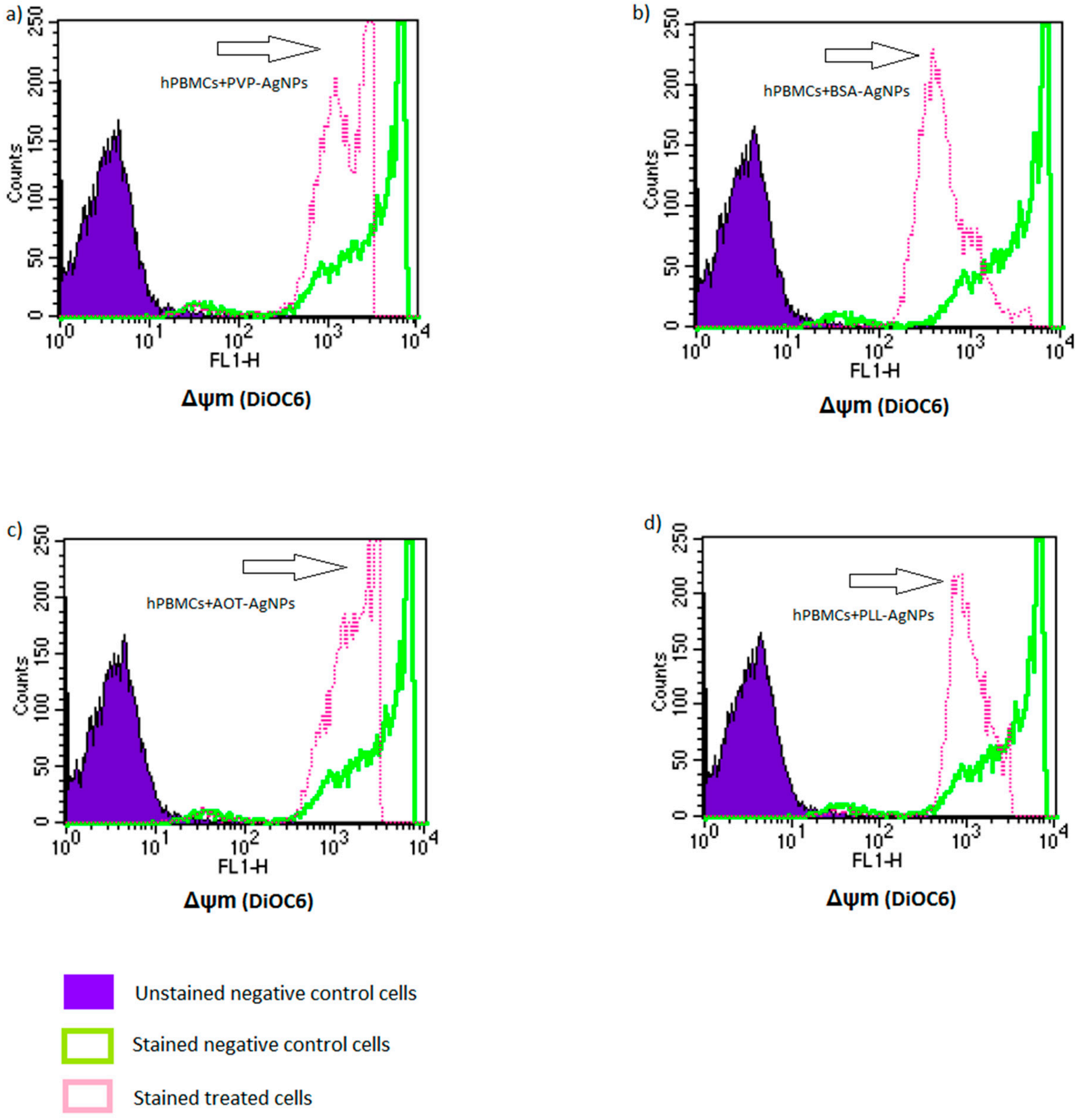

Figure S4. FCM histogram analysis of changes in mitochondrial membrane potential in cells stained with $DiOC_6$. Cells treated with PVP-AgNPs (a), BSA-AgNPs (b), AOT-AgNPs (c) and PLL-AgNPs (d) show a decrease in fluorescence intensity (FL1-H) compared to stained negative controls.

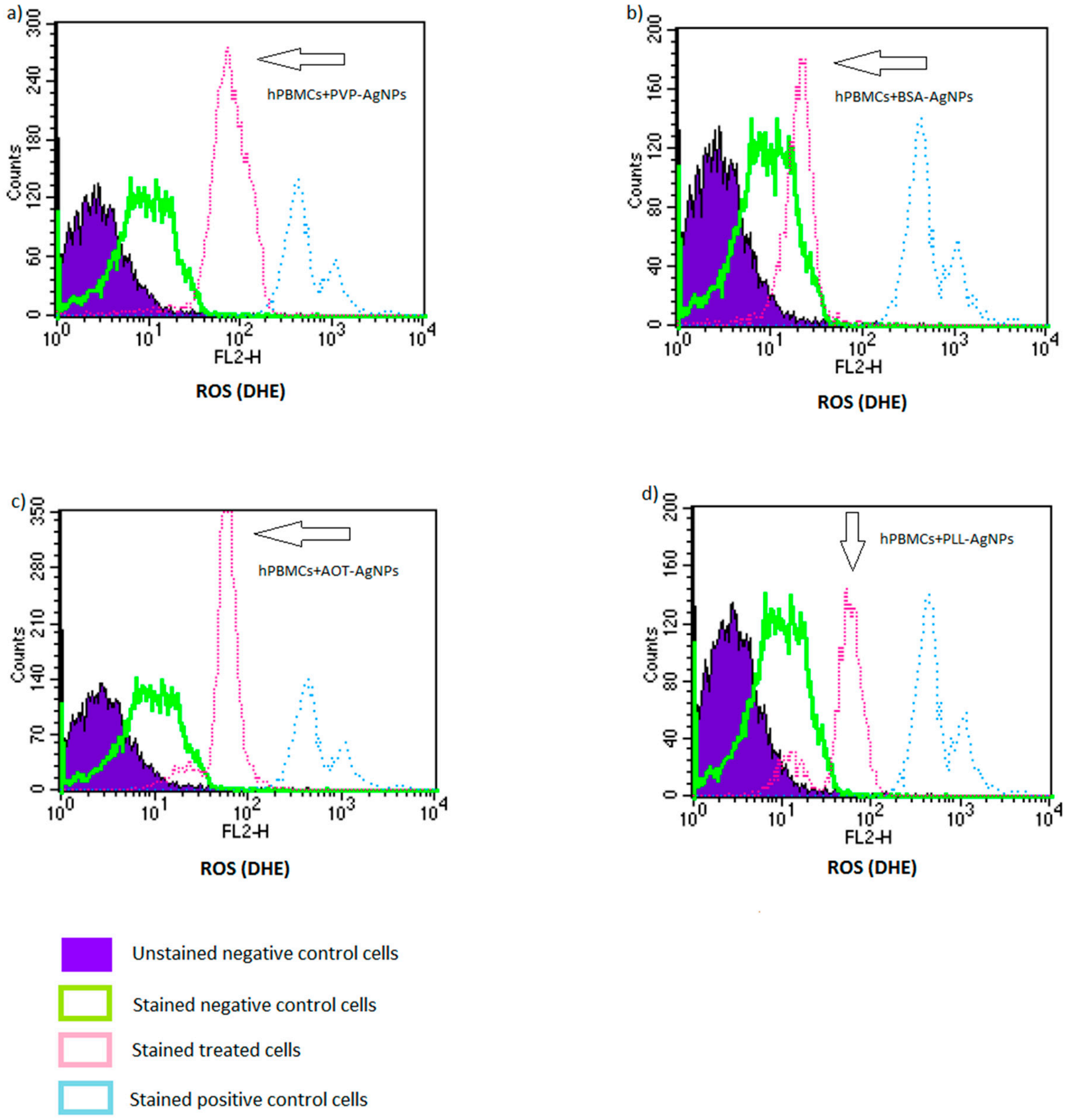

Figure S5. FCM histogram analysis of ROS production in cells stained with DHE. Cells treated with PVP-AgNPs (a), BSA-AgNPs (b), AOT-AgNPs (c) and PLL-AgNPs (d) show an increase in fluorescence intensity (FL1-H) compared to stained negative controls.

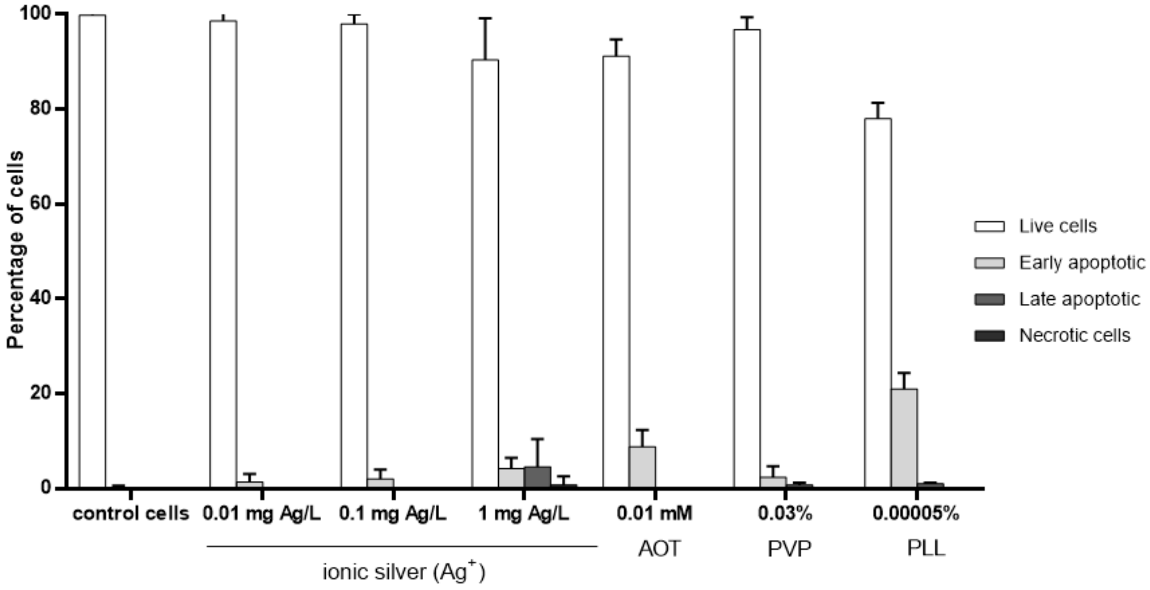

Figure S6. The effect of ionic silver, sodium bis(2-ethylhexyl)-sulfosuccinate (AOT), poly(vinylpyrrolidone) (PVP), and poly-L-lysine (PLL) on the % of live (white columns), early apoptotic (light grey columns), late apoptotic (dark grey columns) and dead (stripped columns) hPBMC after 3 h exposure, determined by flow cytometry after Annexin V/PI staining. Controls were untreated cells. The results are expressed as percentage of controls and given as mean values obtained from six independent experiments. Standard deviations are presented as scale bars.
